# Robert’s Intragastric Alcohol-Induced Gastric Lesion Model as an Escalated General Peripheral and Central Syndrome, Counteracted by the Stable Gastric Pentadecapeptide BPC 157

**DOI:** 10.3390/biomedicines9101300

**Published:** 2021-09-23

**Authors:** Slaven Gojkovic, Ivan Krezic, Hrvoje Vranes, Helena Zizek, Domagoj Drmic, Lovorka Batelja Vuletic, Marija Milavic, Suncana Sikiric, Irma Stilinovic, Paris Simeon, Mario Knezevic, Toni Kolak, Marijan Tepes, Karol Simonji, Sanja Strbe, Nora Nikolac Gabaj, Ivan Barisic, Emma Grace Oreskovic, Eva Lovric, Antonio Kokot, Anita Skrtic, Alenka Boban Blagaic, Sven Seiwerth, Predrag Sikiric

**Affiliations:** 1 Department of Pharmacology, School of Medicine, University of Zagreb, 10000 Zagreb, Croatia; slaven.gojkovic.007@gmail.com (S.G.); ivankrezic94@gmail.com (I.K.); hrvoje.vranes@gmail.com (H.V.); zizekhelena@gmail.com (H.Z.); iddrmic@mef.hr (D.D.); stilinovic.i@gmail.com (I.S.); mariknezevic@gmail.com (M.K.); mtepes@gmail.com (M.T.); strbes@gmail.com (S.S.); inbarisic@gmail.com (I.B.); emmagraceoreskovic@gmail.com (E.G.O.); abblagaic@mef.hr (A.B.B.); 2 Department of Pathology, School of Medicine, University of Zagreb, 10000 Zagreb, Croatia; lbatelja@mef.hr (L.B.V.); marija.milavic@mef.hr (M.M.); suncanasikiric@gmail.com (S.S.); eva.lovric@kb-merkur.hr (E.L.); skrtic.anita@gmail.com (A.S.); sven.seiwerth@mef.hr (S.S.); 3 Department of Endodontics and Restorative Dentistry, School of Dental Medicine, University of Zagreb, 10000 Zagreb, Croatia; simeon@sfzg.hr; 4 Department of Surgery, School of Medicine, University of Zagreb, 10000 Zagreb, Croatia; tkolak@kbd.hr; 5 Internal Diseases Clinic, Faculty of Veterinary Medicine, University of Zagreb, 10000 Zagreb, Croatia; ksimonji@vef.hr; 6 Department of Chemistry, University Clinical Hospital Center “Sestre Milosrdnice”, 10000 Zagreb, Croatia; nora.nikolac@gmail.com; 7 Department of Anatomy and Neuroscience, School of Medicine, J.J. Strossmayer University of Osijek, 31000 Osijek, Croatia; antonio.kokot@mefos.hr

**Keywords:** BPC 157, alcohol, cytoprotection, gastric lesion, ECG, brain edema, escalated general peripheral and central syndrome, therapy

## Abstract

We redefined Robert’s prototypical cytoprotection model, namely the intragastric administration of 96% alcohol in order to generate a general peripheral and central syndrome similar to that which occurs when major central or peripheral veins are occluded in animal models. With this redefinition, we used Robert’s model to examine the cytoprotective effects of the stable gastric pentadecapeptide BPC 157. The intragastric administration of alcohol induced gastric lesions, intracranial (superior sagittal sinus) hypertension, severe brain swelling and lesions, portal and vena caval hypertension, aortal hypotension, severe thrombosis, inferior vena cava and superior mesenteric vein congestion, azygos vein failure (as a failed collateral pathway), electrocardiogram disturbances, and heart, lung, liver and kidney lesions. The use of BPC 157 therapy (10 µg/kg or 10 ng/kg given intraperitoneally 1 min after alcohol) counteracted these deficits rapidly. Specifically, BPC 157 reversed brain swelling and superior mesenteric vein and inferior vena caval congestion, and helped the azygos vein to recover, which improved the collateral blood flow pathway. Microscopically, BPC 157 counteracted brain (i.e., intracerebral hemorrhage with degenerative changes of cerebral and cerebellar neurons), heart (acute subendocardial infarct), lung (parenchymal hemorrhage), liver (congestion), kidney (congestion) and gastrointestinal (epithelium loss, hemorrhagic gastritis) lesions. In addition, this may have taken place along with the activation of specific molecular pathways. In conclusion, these findings clarify and extend the theory of cytoprotection, offer an approach to its practical application, and establish BPC 157 as a prospective cytoprotective treatment.

## 1. Introduction

This study attempts to clarify and extend the theory of cytoprotection, offer an approach to its practical application, and establish a prospective cytoprotective treatment. Cytoprotection is one of the most important concepts in gastroenterology and pharmacology [1,2,3]. The term and concept of cytoprotection were pioneered by Robert et al. in 1979 in response to the noxious gastric effect of the application of intragastric alcohol and non-steroidal anti-inflammatory drugs (NSAIDs) [1,2,3]. Robert’s breakthrough in gastroenterology was cytoprotection, which he defined in general terms as the counteraction of the lesions arising from direct detrimental contact with a noxious agent. The specific model involves the counteraction of intragastric alcohol-induced gastric lesions [1,2,3].

Our conceptual reconstruction [4,5,6,7] took Robert’s prototypical model of intragastric alcohol-induced gastric lesions [1,2,3] as its starting-point. We revealed that intragastric alcohol induces multiorgan pathology and blood pressure disturbances due to endothelial failure, generalized thrombosis and vessel occlusion [4,5,6,7,8]. These findings support and reinforce the original proposition that the epithelium/endothelium is involved in cytoprotection and remains the target of cytoprotective therapy [1,8]. We have theorized and reinforced the observation that the stable gastric pentadecapeptide BPC 157 has cytoprotective effects for the epithelium/endothelium against lesions induced by intragastric alcohol [4] and NSAIDs [9] due to the modulation of prostaglandins [9], the nitric oxide (NO) system [10] and the effects of vascular recovery on collateral pathways (for a review, see [5,6]). Overall, BPC 157 counteracts in this way the syndromes induced in rat models, in which major peripheral or central vessels were occluded [11,12,13,14,15,16,17]. Thus, we assumed that intragastric alcohol administration causes similar widespread dysfunction to that observed in rats after the occlusion of peripheral [11,12,13,14,15,16] and central [17] vessels. These peripheral and central deficits include severe gastric lesions [4], intracranial (superior sagittal sinus) hypertension, brain swelling and lesions, portal and vena caval hypertension, aortal hypotension, peripheral and central thrombosis, inferior and superior vena caval congestion, azygos vein failure, electrocardiogram (ECG) disturbances, and heart, lung, liver and kidney lesions. This syndrome could have prominent screening potential for the cytoprotective activity of anti-ulcer agents.

However, this eventuality has not been investigated, nor appropriately combined with Robert’s cytoprotection [1,2,3,8], in the determination of whether BPC 157 therapy could counteract the peripheral and central deficits mentioned above.

Conceptually, screening the ability of compounds to modulate multiorgan pathology is consistent with Robert’s original proposal, which examined direct injury to the epithelial cells in the stomach by various noxious agents (i.e., intragastric absolute alcohol) [1,2,3] and lead to the smooth extension of cytoprotective effects to other epithelia (i.e., wound-healing effects). In other words, a cytoprotective agent would be released and have a marked range of protective effects, including organ protection [1,2,3]. The multiorgan pathology of intragastric alcohol inducement has been shown in experimental animals [18,19,20] and patients [21,22,23,24].

The exposure of the stomach mucosa to 100% ethanol for as little as 30 s leads to endothelial failure, generalized thrombosis and vessel occlusion in a multiorgan pathology screening model, with abundant thrombi in superficial capillaries, diffuse vascular stasis and endothelial lesions [25]. Cytoprotective agents would follow Szabo’s maxim of endothelium protection → epithelium protection [7,25,26,27,28,29,30]. Therefore, we suggest that intragastric alcohol-induced endothelial lesions exhibit Virchow’s triad—endothelial lesions, hypercoagulability and stasis—which should be counteracted by therapy provided inside and outside the stomach and gastrointestinal tract [5,6]. Indeed, BPC 157 treatment ameliorates Virchow’s triad: it maintains the stomach’s endothelial integrity against noxious agents [31]; prevents and reverses the development of thrombosis after abdominal aorta anastomosis [32], major vein occlusion [11,12,14,15,16,17], artery occlusion [13] or combined vein and artery occlusion [16]; maintains thrombocyte function, without interfering with coagulation pathways [33,34,35]; and evidently counteracts the syndrome induced by major vessel occlusion [11,12,13,14,15,16,17].

For the reliable counteraction of intragastric absolute alcohol application leading to a full syndrome like that described in rats, with the occlusion of major vessels [11,12,13,14,15,16,17], BPC 157 served as a ‘bypassing key’ that can be applied to the injurious occlusion. This effect has been observed in inferior vena caval syndrome [11], the Pringle maneuver (portal triad temporary occlusion), ischemia/reperfusion [12], Budd–Chiari syndrome (suprahepatic inferior vena cava occlusion) [14], superior mesenteric artery occlusion syndrome [13,16], superior mesenteric vein occlusion syndrome [15,16], superior mesenteric artery and vein occlusion syndrome [16], and superior sagittal sinus occlusion syndrome [17]. The rapid vessel recruitment and the activation of collateral pathways (i.e., the left ovarian vein [11], inferior mesenteric vein [12,14], azygos vein [14], inferior and superior pancreaticoduodenal veins and pyloric vein [15], the inferior anterior pancreaticoduodenal artery and inferior mesenteric artery [13,16]) re-establish blood flow, thereby compensating for vessel occlusion [11,12,13,14,15,16,17,36,37,38,39]. BPC 157 therapy attenuated/eliminated the consequences of Budd–Chiari syndrome, including the activation of a left superior vena cava-azygos vein-inferior vena cava shunt to avoid the suprahepatic occlusion of the inferior vena cava, which allowed the survival of animals [14]. In addition, heart dysfunction, lung lesions (i.e., time-dependent and time-independent features that can be exudative phase features of acute respiratory distress syndrome [ARDS]), liver failure, gastrointestinal lesions, widespread arterial and venous thrombosis, severe portal and vena caval hypertension and aortal hypotension were all counteracted [14]. A similar syndrome, including intracranial hypertension, was noted with the occlusion of the superior sagittal sinus [17]. BPC 157 therapy counteracted intracranial (superior sagittal sinus) hypertension, brain swelling and lesions in a similar way to the previous peripheral vessel occlusion studies [11,12,13,14,15,16,17]. Hence, it appears that the maxim, endothelium maintenance → epithelium maintenance [1,8], can be upgraded to endothelium maintenance → epithelium maintenance = blood vessel recruitment and activation (‘running’) towards the site of injury, also described as bypassing occlusions via alternative pathways [5,6]. Furthermore, there is evidence that after clamping the common carotid arteries, BPC 157 administered during reperfusion counteracted stroke and both early and delayed neural hippocampal damage, lead to full functional recovery (based on the Morris water maze test, inclined beam-walking test and lateral push test) [40]. Likewise, BPC 157 counteracted retinal ischemia induced by one retrobulbar application of the nitric oxide synthase (NOS) blocker L-NAME [41]. Evidently, these effects support the role of BPC 157 in the gut–brain axis and central effects [42,43], a relevant point regarding the possible counteraction against intragastric alcohol-induced multiorgan pathology.

BPC 157 was originally described as an anti-ulcer peptide that is stable in human gastric juice for more than 24 h [44]. It is advantageous in wound-healing [7,45], and its effects are distinct from those of the standard angiogenic growth factors that rapidly degrade in human gastric juice [7,44,45]. BPC 157 interacts with several molecular pathways [40,46,47,48,49,50,51,52,53,54], exerts modulatory effects on the NO system [10], vasomotor tone and the activation of the Src–Caveolin-1–endothelial nitric oxide synthase (eNOS) pathway [48], stabilizes cellular junctions [46], and scavenges free radicals [55,56,57], in addition to the benefits it has shown in vascular occlusion studies [11,12,13,15,16,36,37,38,39].

The present study is the first to examine the treatment options for the full occlusive syndrome, including peripheral and central deficits similar to those described in rats with major vessel occlusion, that occurs following the intragastric administration of absolute alcohol (epithelial and endothelial injuries and thrombosis), as in Robert’s prototypical cytoprotective model [11,12,13,14,15,16,17]. We aimed to determine whether the administration of BPC 157 activated bypassing collateral pathways to provide cytoprotection. We hypothesized that, in accordance with its previous therapeutic effect in occlusive syndromes [11,12,13,14,15,16,17], the administration of BPC 157 counteracts gastric lesions, peripheral (portal and vena caval hypertension, aortal hypotension) and central (i.e., brain swelling and intracranial hypertension) disturbances and organ lesions. In addition, this may take place along with the activation of the specific molecular pathways, i.e., *eNOS*, *mTOR* and *VGFEa*, known to interact with BPC 157 and the administration of alcohol [7,10,58,59].

Finally, our findings could demonstrate the benefits of this model for screening potentially cytoprotective compounds.

## 2. Materials and Methods

### 2.1. Animals

This study was conducted with 12-week-old, 200 g male albino Wistar rats, randomly assigned at six rats/group/interval. The rats were bred in-house at the Pharmacology Animal Facility, School of Medicine, Zagreb, Croatia. The animal facility was registered by the Directorate of Veterinary (Reg. No: HR-POK-007). The laboratory rats were acclimated for 5 days and then randomly assigned to their respective treatment groups. They were housed in polycarbonate cages under conventional laboratory conditions at 20–24 °C, with a relative humidity of 40–70% and a noise level of 60 dB. Each cage was identified by dates, the study number, the group, the dose and the number of the animal. Fluorescent lighting provided illumination 12 h per day. A standard good laboratory practice (GLP) diet and fresh water were provided *ad libitum*. The animals’ care was in compliance with the standard operating procedures (SOPs) of the Pharmacology Animal Facility and the European Convention for the Protection of Vertebrate Animals used for Experimental and other Scientific Purposes (ETS 123).

This study was approved by the local Ethics Committee. The ethical principles of the study complied with the European Directive 010/63/E, the Law on Amendments to the Animal Protection Act (Official Gazette 37/13), the Animal Protection Act (Official Gazette 135/06), the Ordinance on the protection of animals used for scientific purposes (Official Gazette 55/13), the Federation of European Laboratory Animal Science Association (FELASA)’s recommendations and the recommendations of the Ethics Committee of the School of Medicine, University of Zagreb. The experiments were assessed by observers blinded to the treatment.

### 2.2. Drugs and Experimental Protocol

The medication was administered as described in previous studies [11,12,13,14,15,16,17], without the use of a carrier or a peptidase inhibitor for stable gastric pentadecapeptide BPC 157, a partial sequence of the human gastric juice protein BPC, which is freely soluble in water at pH 7.0 and in saline. The BPC 157 (GEPPPGKPADDAGLV, with a molecular weight 1419 Da; Diagen, Slovenia) was prepared as a peptide with 99% high-performance liquid chromatography (HPLC) purity, with the peptide 1-des-Gly as the main impurity. The dose and application regimens were the same as described in previous studies [11,12,13,14,15,16,17]. Briefly, deeply anaesthetized rats received 1 mL of 96% alcohol delivered intragastrically. One minute after the injection, BPC 157 (10 µg/kg or 10 ng/kg), or an equal volume of saline (5 mL/kg), was administered intraperitoneally. The rats were euthanized 1, 5, 15 or 30 min following the injection.

### 2.3. Gross Lesion Presentation

The gross lesions were recorded in deeply anaesthetized laparatomized rats, with a camera attached to a VMS-004 Discovery Deluxe USB microscope (Veho, Dayton, OH, USA). Hemorrhagic lesions in the stomach were assessed as the percentage of the total area of the glandular stomach at 1 min after alcohol administration and following medication, at 1, 5, 15 and 30 min; the rats were then euthanized. The other investigated features included the gross presentation of the brain, the superior mesenteric vein, the inferior vena cava and the heart, as well as a thrombus assessment.

### 2.4. Assessment of the Change in the Brain, Vein or Heart Volume Proportional to the Change in the Brain, Vein or Heart’s Surface Area

As described in previous studies [13,14,15,16,17], we recorded the presentation of the brain, peripheral veins (superior mesenteric vein, inferior vena cava and azygos vein) and heart of deeply anaesthetized rats that had undergone laparotomy or complete calvariectomy, using a camera attached to a VMS-004 Discovery Deluxe USB microscope (Veho, Dayton, OH, USA). This endeavor was performed before intragastric alcohol administration to the healthy rats and then 1 min after the introduction of 1 mL of 96% alcohol in the stomach, before therapy initiation (1 min injury time), and 1, 5, 15 and 30 min after therapy (saline or BPC 157); the rats were then euthanized. The border of the brain, vein or heart in the photographs was marked using ImageJ software (National Institutes of Health, Bethesda, MD, USA). Next, the surface area (in pixels) of the brain, vein, or heart was measured using a measuring function. This was performed with the brain, vein or heart photographs at each of the above-mentioned time-points for both the control and alcohol-treated animals. In the rats administered alcohol, the brain, vein or heart area before application was marked as 100%, and the ratio of each subsequent brain, vein or heart area relative to the first area was calculated as ($\frac{A_{2}}{A_{1}}$). Starting from the square-cube law Equations (1) and (2), an equation for the change in the brain, vein or heart volume proportional to the change in the brain, vein or heart surface area (6) was derived. For expressions (1)–(5), any arbitrary one-dimensional length on the photograph was defined (e.g., the rostro-caudal length of the brain, or any arbitrary length of a vein or the heart). It was used only to define the one-dimensional proportion (*l*_2_/*l*_1_) between two observed brains, veins or hearts and as an inter-factor (and therefore not measured) for deriving the final expression (6). The procedure was as follows:

(1) $$A_{2}=A_{1}\times {\left(\frac{l_{2}}{l_{1}}\right)}^{2}$$

square-cube law,

(2) $$V_{2}=V_{1}\times {\left(\frac{l_{2}}{l_{1}}\right)}^{3}$$

square-cube law,

(3) $$\frac{A_{2}}{A_{1}}={\left(\frac{l_{2}}{l_{1}}\right)}^{2}$$

from (1), after dividing both sides by A_1_,

(4) $$\frac{l_{2}}{l_{1}}=\sqrt{\frac{A_{2}}{A_{1}}}$$

from (3), after taking the square root of both sides,

(5) $$\frac{V_{2}}{V_{1}}={\left(\frac{l_{2}}{l_{1}}\right)}^{3}$$

from (*2*), after dividing both sides by V_1_ and

(6) $$\frac{V_{2}}{V_{1}}={\left(\sqrt{\frac{A_{2}}{A_{1}}}\right)}^{3}$$

after incorporating (4) into (5).

This measuring procedure, followed by the calculation of the volume ratios, was performed separately for the brain, veins and heart.

Brain swelling was recorded in separate rats 15 min after complete calvariectomy. Briefly, 6 burr holes were drilled in three horizontal lines, all of them medial to the superior temporal lines and temporalis muscle attachments. The two rostral burr holes were placed just basally from the posterior interocular line, the two basal burr holes were placed just rostrally to the lambdoid suture (and the transverse sinuses) on both sides, respectively, and the middle two burr holes were placed in the line between the basal and rostral burr holes.

### 2.5. Thrombus Assessment

After euthanasia, the superior sagittal sinus, the portal vein, the inferior vena cava, the superior mesenteric vein, the superior mesenteric artery, the hepatic artery and the abdominal aorta were removed from the rats, and the clots were weighed [11,12,13,14,15,16,17].

### 2.6. Superior Sagittal Sinus, Portal Vein, Vena Caval and Abdominal Aortal Pressure Recording

Recordings were made in the deeply anaesthetized rats with a cannula (BD Neoflon™ Cannula, BD Switzerland, Eysins, Switzerland), connected to a pressure transducer (78534C MONITOR/TERMINAL; Hewlett Packard, Palo Alto, CA, USA), which was inserted into the superior sagittal sinus, the portal vein, the inferior vena cava and the abdominal aorta at the level of the bifurcation before the administration of alcohol 1 min after alcohol application and 1, 5, 15 and 30 min after therapy (saline or BPC 157). Each recording lasted 1 min. For the superior sagittal sinus pressure recording, we made a single burr hole in the rostral part of the sagittal suture, above the superior sagittal sinus, and cannulated the anterior portion of the superior sagittal sinus using Braun intravenous cannulas. We then laparatomized the rats in order to record portal vein, inferior vena caval and abdominal aortal pressure.

Of note, the normal rats exhibited a superior sagittal sinus pressure of −24 to −27 mmHg and a portal pressure of 3–5 mmHg, which was similar to that of the inferior vena cava, although it was at least 1 mmHg higher in the portal vein. By contrast, the abdominal aorta blood pressure was 100–120 mm Hg at the level of the bifurcation [11,12,13,14,15,16,17].

### 2.7. ECG Recording

ECGs were recorded continuously in the deeply anaesthetized rats for all three main leads by positioning stainless steel electrodes on all four limbs using an ECG monitor with a 2090 programmer (Medtronic, Minneapolis, MN, USA) connected to a Waverunner LT342 digital oscilloscope (LeCroy, Spring Valley Village, NY, USA) at 15 min, 24 h or 48 h after ligation. This arrangement enabled precise recordings, measurements and analysis of the ECG parameters at the level of the bifurcation [11,12,13,14,15,16,17].

### 2.8. Microscopy

#### 2.8.1. Tissue Preparation

The stomach (from grossly intact tissue), brain, lungs, liver, kidneys and heart were rapidly removed and fixed in 10% neutral buffered formalin at room temperature for 24 h. Tissue blocks were embedded in paraffin, sectioned at 4 μm, stained with hematoxylin and eosin (H&E) and evaluated by light microscopy using semiquantitative scoring.

#### 2.8.2. Brain Histology

As described [13,14,15,16,17], two coronal sections of each brain were prepared according to NTP-7, Levels 3 and 6, considering the neuroanatomic subsites present in certain brain sections [60]. At NTP-7 Level 3, we observed the areas of the fronto-parietal cortex and hippocampus. At NTP-7 Level 6, we analyzed the cerebellar cortex. Brain coronal blocks were embedded in paraffin, sectioned at 1 μm, stained with H&E and evaluated by light microscopy using neuropathological scoring [61]. The number of dark neurons in the temporal cortex and in the hippocampus was counted in an area of 26,406 µm^2^, with 80 measurements for each sample of cortex and hippocampus. The dark neurons were subjected to hypoxia; they featured eosinophilic cytoplasm, pyknotic nuclei and loss of Nissl substance.

#### 2.8.3. Lung Histology

We used a scoring system to grade the degree of lung injury, including observations of focal thickening of the alveolar membranes, congestion, pulmonary edema, intra-alveolar hemorrhage, interstitial neutrophil infiltration and intra-alveolar neutrophil infiltration. Each feature was assigned a score from 0 to 3, based on its absence (0), or presence to a mild (1), moderate (2) or severe (3) degree. A final histology score was determined [13,14,15,16,17,62].

#### 2.8.4. Renal, Liver and Heart Histology

The assessment of renal injury was based on the degeneration of Bowman’s space, glomeruli and proximal and distal tubules, vascular congestion, and interstitial edema. The criteria for liver injury were the vacuolization of hepatocytes and pyknotic hepatocyte nuclei, the activation of Kupffer cells and the enlargement of sinusoids. Each specimen was scored using a scale from 0 to 3 (0, none; 1, mild; 2, moderate; 3, severe) for each criterion [13,14,15,16,17,63]. The myocardium was graded for the severity of necrosis exhibited in the ventricles. The mean value of their scores is presented. The pathological criteria for grading the severity of necrosis were: score 1 (mild), one or two small foci; score 2 (slight), several small foci; score 3 (moderate), multiple small foci or several large foci; score 4 (severe), multiple large foci or diffuse area of necrosis [13,14,15,16,17,64].

### 2.9. Gene Expression Analysis

To illustrate the possible involvement of pathways, gene expression analysis assessment (Table 1) was performed on the rats at 5 min after intragastric alcohol administration, and intraperitoneal administration of 5 mL/kg of saline or 10 ng/kg of BPC 157.

The total RNA was extracted from different tissues, rapidly dissected, snap-frozen in liquid nitrogen and stored at −80 °C. The tissues were homogenized using a Bio-Gen PRO200 homogenizer (PRO Scientific, Willenbrock Rd, Oxford, CT, USA) in 1000 μL of TRIzol (Invitrogen, Thermo Fisher Scientific, Waltham, MA, USA), and RNA extraction was performed using a TRIzol-based reagent method according to the manufacturer’s instructions.

After RNA extraction, quantification was performed with DeNovix DS-11 Spectrophotometer (DeNovix Inc., Wilmington, DE, USA).

A High Capacity cDNA Reverse Transcription Kit (Applied Biosystems, Thermo Fisher Scientific, Waltham, MA, USA) was used to perform reverse transcription following the manufacturer’s instructions and using a ProFlex PCR System machine (Applied Biosystems, Thermo Fisher Scientific, Waltham, MA, USA).

TaqMan Gene Expression Assays (Applied Biosystems, Thermo Fisher Scientific, Waltham, MA, USA) with a TaqMan Gene Expression Master Mix were used for the gene expression analysis of selected genes (Table 1). Quantitative PCR was carried out in duplicate for every sample. A Cobas z 480 instrument (Hoffmann-La Roche Ltd., Basel, Switzerland) was used to perform qPCR under the following conditions: 2 min at 50 °C, 10 min at 95 °C, 45 cycles of 15 sec at 95 °C and 1 min at 60 °C.

*Actb* was chosen as a reference gene for the normalization of the *Nos3*, *Mtor and Vegfa* gene expression data.

The difference in gene expression between the treated and non-treated samples was analyzed using the formula 2^−ΔΔCt^, where ΔΔCt is the difference between the ΔCt of the treated sample and the ΔCt of the non-treated sample. The results were expressed as fold change and as percentages. Fold change values lower then 70% indicated decreased gene expression in BPC-157-treated animals (downregulation), fold change values between 70% and 130% were considered as biological variability (no change in gene expression), and fold change values higher then 130% indicated increased gene expression in BPC-157-treated animals (upregulation).

### 2.10. Statistical Analysis

The statistical analysis was performed by using the parametric one-way analysis of variance (ANOVA) with the post hoc Newman–Keuls test or the non-parametric Kruskal–Wallis test, followed by the Mann–Whitney *U* test to compare the groups. The values were presented as the mean ± standard deviation (SD) or as the minimum, median and maximum. To compare the frequency difference between the groups, the chi-square test or Fischer’s exact test was used. A value of *p* < 0.05 was considered statistically significant.

## 3. Results

We found that an assault on the stomach—96% alcohol administered intragastrically—produced gastric lesions and rapidly escalated to a generalized syndrome with peripheral and central deficits similar to those that have been observed when major vessels are occluded in rats [11,12,13,14,15,16,17]. It was completely counteracted by BPC 157 therapy, using either the ng/kg or µg/kg regimen. A likely interpretation is that BPC 157 administration may have extended the innate cytoprotective effect, particularly the vascular effect that is rapidly activated by intragastric alcohol exposure. The beneficial effects then extended peripherally and centrally. 

### 3.1. Stomach

**Figure 1:**
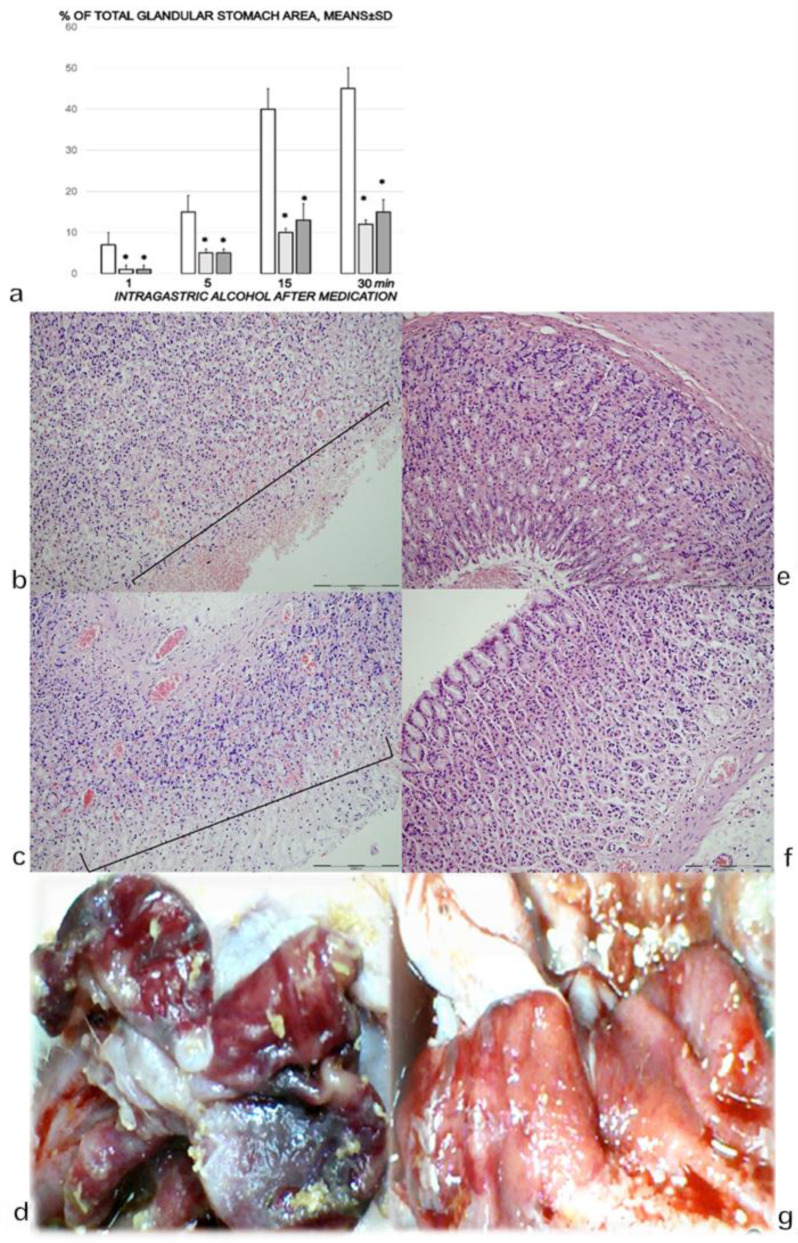
(**a**). Stomach lesions (% of total glandular stomach area). Controls (white bars) with the administered 1 mL of the 96% alcohol into the stomach showed marked hemorrhagic lesions within the stomach, with an ascending course from the first minute until the end (1, 5, 15, and 30 min following therapy, which was administered at 1 min following the introduction of 96% alcohol in the stomach). The course was markedly counteracted with BPC 157 therapy (10 µg (light gray bars) or 10 ng/kg (dark gray bars) IP). Six rats/group/interval. * *p* ˂ 0.05, at least vs. the corresponding control. (**b**,**c**). Histological (HE, ×100, scale bar 200 µm) presentation of stomach mucosa. One mL of 96% alcohol was applied directly into the rats’ stomachs, and saline 5 mL/kg IP was administered at 1 min following the introduction of 96% alcohol in the stomach. Microscopically (specimens taken at the areas grossly intact), the control rats presented marked congestion and erosive gastritis, as illustrated in (**b**) (1 min; mucosal surface erosions marked), and **c** (30 min; mucosal surface erosions) with full brace). (**d**). Alcohol lesions in controls at 15 min (camera attached to a VMS-004 Discovery Deluxe USB microscope (Veho, Dayton, OH, USA)). (**e**,**f**). Histological (HE, ×100, scale bar 200 µm) presentation of stomach mucosa. One mL of 96% alcohol was applied directly into the rats’ stomachs, and BPC 157 therapy (10 µg or 10 ng/kg) IP was administered at 1 min following the introduction of 96% alcohol in the stomach, with marked attenuation at 1 min (**e**), and at 30 min (**f**). (**g**). Alcohol lesions markedly attenuated in BPC-157-treated rats at 15 min (camera attached to a VMS-004 Discovery Deluxe USB microscope (Veho, Dayton, OH, USA)).

Alcohol insult to the stomach produced large gross hemorrhagic lesions and severe pathology in the stomach (i.e., mucosal surface erosion, even in areas that were macroscopically intact) (Figure 1a–d). In the stomachs of the rats that received BPC 157, the lesions were markedly attenuated, and in the areas that were macroscopically intact, the microscopic presentation showed no gross changes and no microscopic congestion of the stomach mucosa (Figure 1a–g).

### 3.2. Liver

**Figure 2:**
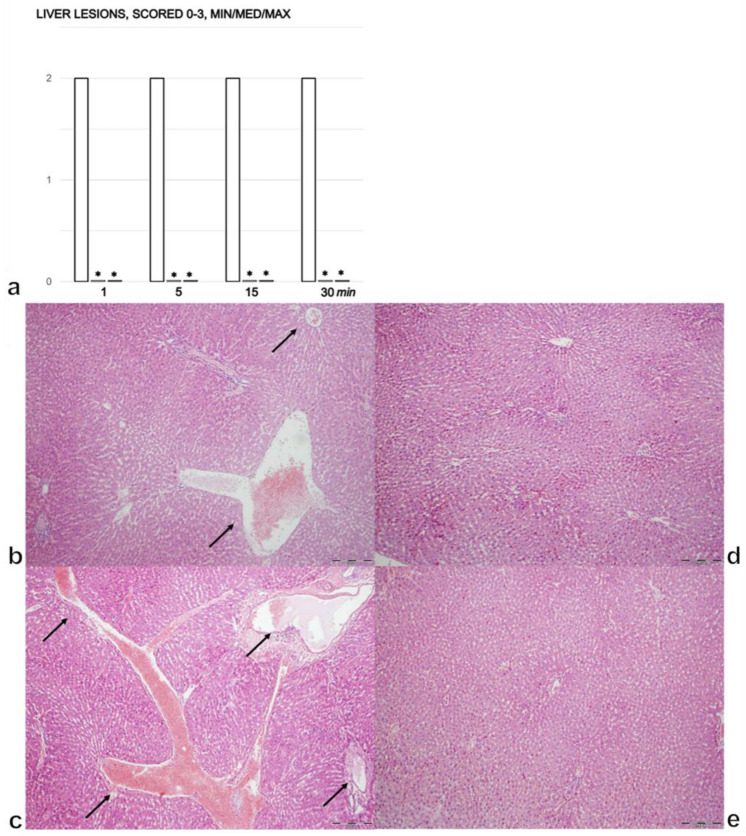
(**a**). Liver lesions, scored 0–3, assessed in the rats with 1 mL of the 96% alcohol introduced in their stomachs, at 1, 5, 15, and 30 min following therapy. Therapy (BPC 157 (10 µg (light gray bars) or 10 ng/kg (dark gray bars); saline (5 mL/kg (control, white bars)), IP) was administered at 1 min following the introduction of 96% alcohol in the stomach. The course was markedly counteracted with BPC 157 therapy. Six rats/group/interval. * *p* ˂ 0.05 at least vs. the corresponding control. (**b**–**e**). Histology of the liver parenchyma (**b**–**e**). After the immediate period following the introduction of 96% alcohol in the stomach, the controls presented with congestion (dilatation of central veins, sinusoids and blood vessels in portal tracts), which progressed in the liver tissue (**b** (1 min), **c** (30 min), arrows). The rats treated with BPC 157 showed no changes in their liver tissues (**d** (1 min), **e** (30 min)); (HE, ×100, scale bar 200 µm). The pictures obtained using BPC 157 10 ng/kg IP are representative of the observations for both doses.

Within 1 min of intragastric alcohol administration, there was moderate congestion in the liver (Figure 2a,b). After 5 min, there was further progression of liver congestion. After 15 min, we noted prominent congestion. After 30 min, there was prominent congestion and a ballooning of hepatocytes in zone 3 of the liver lobules (Figure 2a,c). For rats the that received BPC 157, in the 30 min post-injury period, there were no changes found in the liver (Figure 2a,d,e).

### 3.3. Kidney

**Figure 3:**
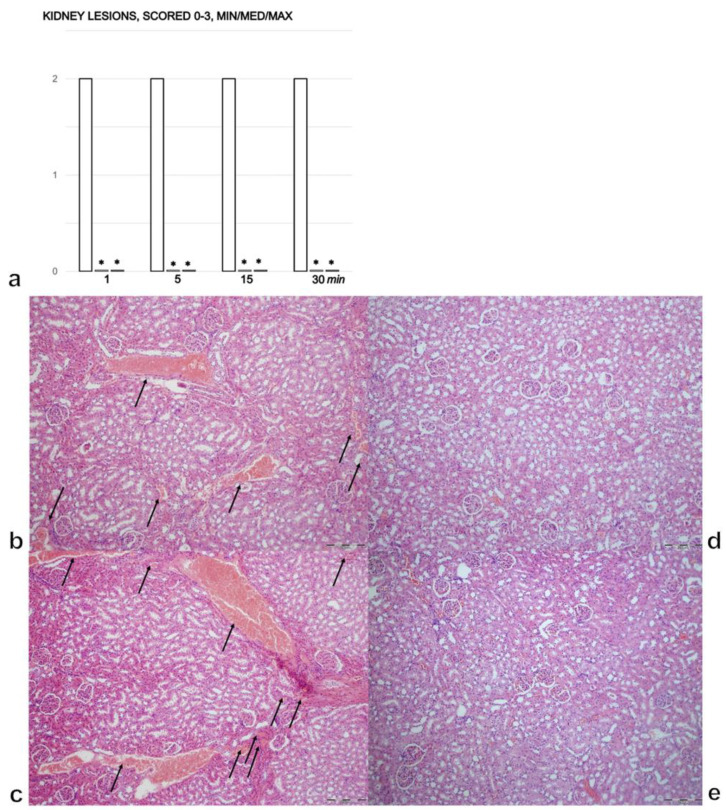
(**a**). Kidney lesions, scored 0–3, assessed in the rats with 1 mL of 96% alcohol introduced in their stomachs, at 1, 5, 15, and 30 min following therapy. Therapy (BPC 157 (10 µg (light gray bars) or 10 ng/kg (dark gray bars); saline (5 mL/kg (control, white bars)), IP) was administered at 1 min following the introduction of 96% alcohol in the stomach. The course was markedly counteracted with BPC 157 therapy. Six rats/group/interval. * *p* ˂ 0.05 at least vs. the corresponding control. (**b**–**e**). Histology of the renal parenchyma. After the immediate period following the introduction of 96% alcohol in the stomach, the controls presented with congestion, and its progression in the renal tissues presented with dilated and congested small, medium and large blood vessels, as well as glomeruli (**b** (1 min), **c** (30 min), arrows). The rats treated with BPC 157 showed no changes in their kidneys (**d** (1 min), **e** (30 min)); (HE, ×100, scale bar 200 µm). The pictures obtained using BPC 157 10 ng/kg IP are representative of the observations for both doses.

Alcohol insult to the stomach led to kidney lesions within 1 min, moderate congestion and progression after 5 min and prominent congestion after 15 and 30 min (Figure 3a–c). In the rats treated with BPC 157, there were no changes in the kidneys 30 min after treatment (Figure 3a,d,e).

### 3.4. Lung

**Figure 4:**
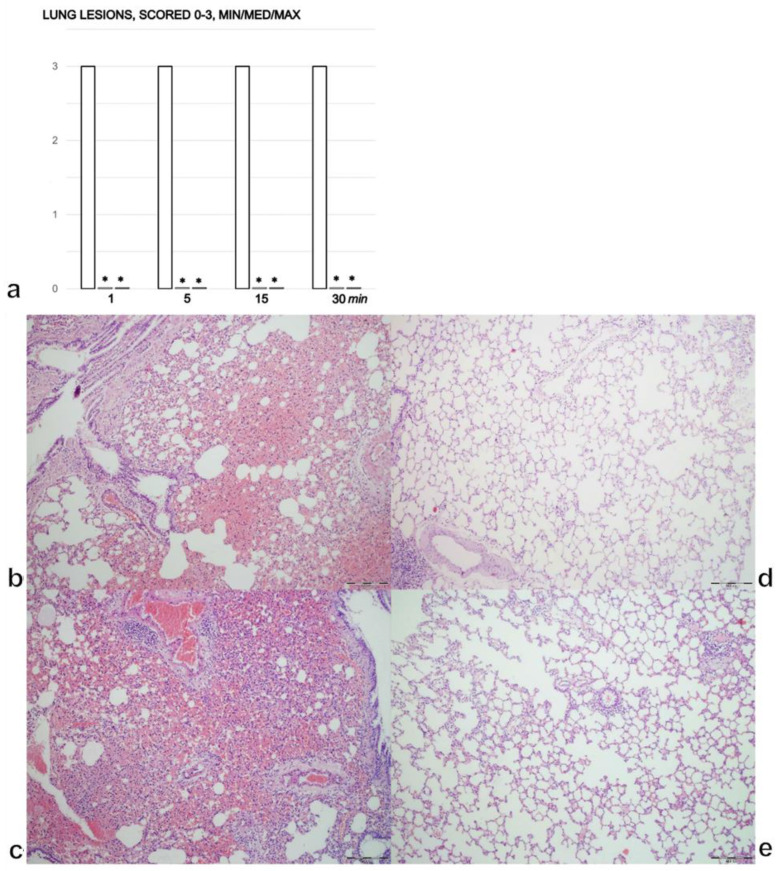
(**a**) Lung lesions, scored 0–3, assessed in the rats with 1 mL of 96% alcohol introduced in their stomachs, at 1, 5, 15, and 30 min following therapy. Therapy (BPC 157 (10 µg (light gray bars) or 10 ng/kg (dark gray bars); saline (5 mL/kg (control, white bars)), IP) was administered at 1 min following the introduction of 96% alcohol in the stomach. The course was markedly counteracted with BPC 157 therapy. Six rats/group/interval. * *p* ˂ 0.05 at least vs. corresponding control. (**b**–**e**). Histology of the lung (**b**–**e**). After the immediate period following the introduction of 96% alcohol in the stomach, the controls presented with lung congestion and intraalveolar hemorrhage (**b** (1 min), **c** (30 min)) while the rats treated with BPC 157 presented only mild congestion and no lung hemorrhage (**d** (1 min), **e** (30 min)); (HE, ×100, scale bar 200 µm). The pictures obtained using BPC 157 10 ng/kg IP are representative of the observations for both doses.

In the control rats, there was moderate congestion in the lungs with hemorrhage in the lung parenchyma 1 min after intragastric alcohol administration (Figure 4a,b). After 5 min, there was further progression of tissue congestion with persistent hemorrhage in the lung parenchyma. By 15 and 30 min, we noted prominent congestion and hemorrhage in the lung parenchyma (Figure 4a,c). In the rats treated with BPC 157, there were no changes found in in the lung after 1 and 5 min (Figure 4a,d). After 15 and 30 min, only mild congestion was found in the lung (Figure 4a,e).

### 3.5. Heart

#### Heart Lesions and Dilatation and ECG Recording

**Figure 5:**
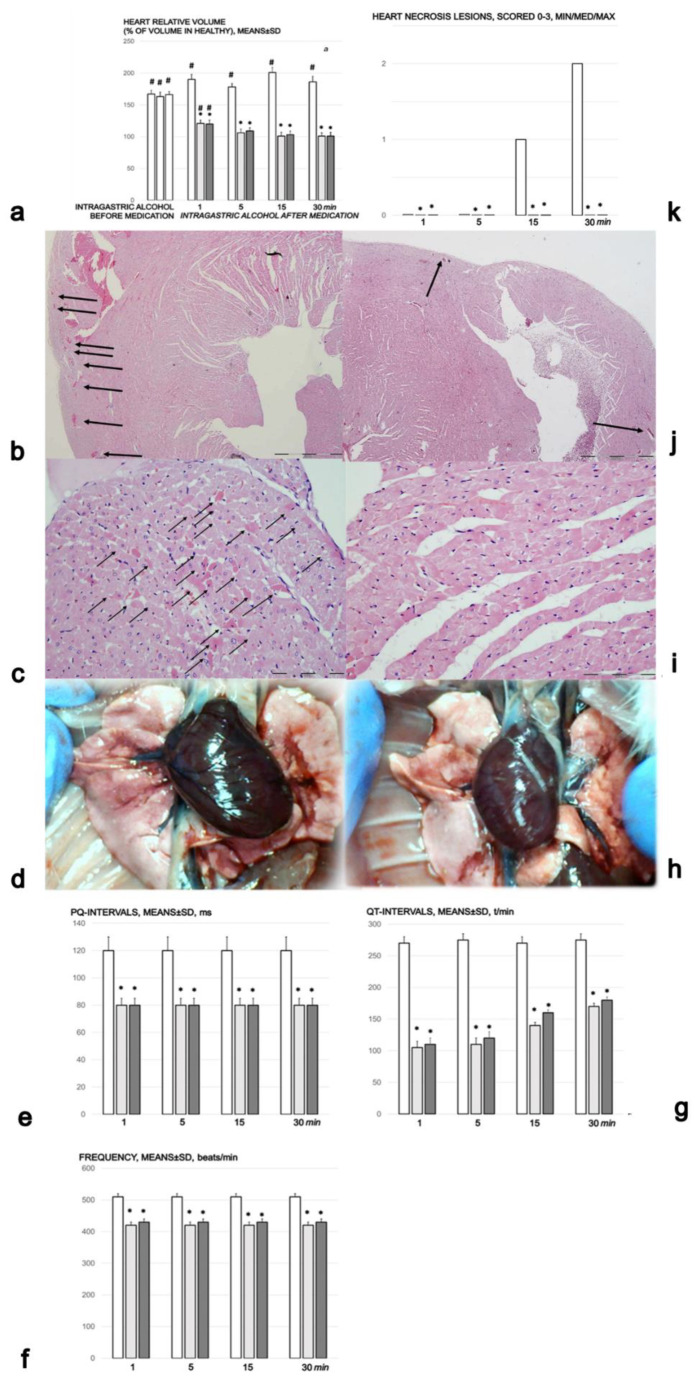
Heart lesions. (**a**) Heart, volume (% of volume in healthy). Controls (white bars) with 1 mL of 96% alcohol introduced in the stomach showed increased heart dilatation from the first minute until the end (1, 5, 15, and 30 min following therapy, which was given at 1 min following 96% alcohol instillation in the stomach (intragastric alcohol before medication)). The course was markedly counteracted with BPC 157 therapy, which consisted of 10 µg (light gray bars) or 10 ng/kg ((dark gray bars) IP). Six rats/group/interval. * *p* ˂ 0.05 at least vs. the corresponding control, # *p* ˂ 0.05 at least vs. normal healthy (100%). (**b**,**c**). Histology of the myocardium in saline-treated rats. Afyer the immediate period following the introduction of 96% alcohol in the stomach, the controls presented with congestion of the myocardium (**b** (1 min), HE, ×100, scale bar 200 µm), progressing to the subendocardial infarct (necrotic myocytes (arrows)) (**c** (30 min), HE, ×200, scale bar 200 µm). (**d**). Gross presentation of the heart in rats that received 1 mL of 96% alcohol introduced directly into the stomach, and saline 5 mL/kg IP, presentation at 30 min thereafter, before euthanasia (camera attached to a VMS-004 Discovery Deluxe USB microscope (Veho, Dayton, OH, USA)). ECG changes (**e**–**g**). PQ- (**a**), QT-intervals (**b**) and frequency (**c**) in the rats with who had 1 mL of 96% alcohol introduced in the stomach, at 1, 5, 15, and 30 min following therapy. Therapy (BPC 157 10 µg (light gray bars) or 10 ng/kg (dark gray bars); saline (5 mL/kg (control, white bars)), IP) was administered at 1 min following the introduction of 96% alcohol in the stomach. The course was markedly counteracted with BPC 157 therapy. Six rats/group/interval. * *p* ˂ 0.05 at least vs. the corresponding control. (**h**). Gross presentation of the heart in the rats that received 1 mL of 96% alcohol introduced directly into the stomach, and BPC 157 10 ng/kg IP at 1 min following alcohol, presentation at 30 min thereafter, before euthanasia (camera attached to a VMS-004 Discovery Deluxe USB microscope (Veho, Dayton, OH, USA)). (**i**,**j**). Histology of the myocardium in rats treated with BPC 157. These rats showed no changes in the heart (**j** (1 min, HE, ×100, scale bar 200 µm), (**i**) (30 min, HE, ×200, scale bar 200 µm)). (**k**). Heart necrosis, scored 0–3, assessed in the rats that had 1 mL of 96% alcohol introduced in the stomach, at 1, 5, 15, and 30 min following therapy. Therapy (BPC 157 10 µg (light gray bars) or 10 ng/kg (dark gray bars); saline (5 mL/kg (control, white bars), IP) was administered at 1 min following the introduction of 96% in the stomach. The course was markedly counteracted with BPC 157 therapy. Six rats/group/interval. * *p* ˂ 0.05 at least vs. the corresponding control. The pictures obtained using BPC 157 10 ng/kg IP are representative of the observations for both doses.

Intragastric alcohol rapidly produced heart dilatation and lesions and ECG disturbances (Figure 5a–k). In the control rats, within 1 min of intragastric alcohol administration, there was moderate congestion in the heart (Figure 5b,k), and this deficit progressed over time (Figure 5c,k). After 5 min, there was tissue congestion and persistent hemorrhage. After 15 min, we noted prominent congestion and, due to low aortic pressure, passive congestion occurred in the myocardium, with acute subendocardial infarct. After 30 min, we found prominent congestion and acute subendocardial infarct in the control group (Figure 5k). The rats treated with BPC 157 showed no changes in the heart (blood vessels marked with arrows) (Figure 5j (1 min) and Figure 5i (30 min)). In the rats treated with BPC 157, there were no gross changes in the heart after 30 min (Figure 5h). BPC 157 counteracted heart dilatation (Figure 5a); this proportional change in the heart surface area was used to assess the development of heart failure.

One minute after the introduction of intragastric, ECG recordings showed marked tachycardia with prolonged PQ and QTc intervals (Figure 5e–g). Furthermore, along with the rapid appearance of heart lesions, the rats presented ST elevation that was highest at the earliest time point (1.3 ± 0.1 at 1 min) and remained high (0.7 ± 0.1) until the end of the experiment (30 min). Treatment with BPC 157 completely counteracted the ST elevation (*p* ˂ 0.05 compared with saline-treated rats). The only abnormality was peaked T waves in the third limb lead at all time points.

### 3.6. Blood Vessels

**Figure 6:**
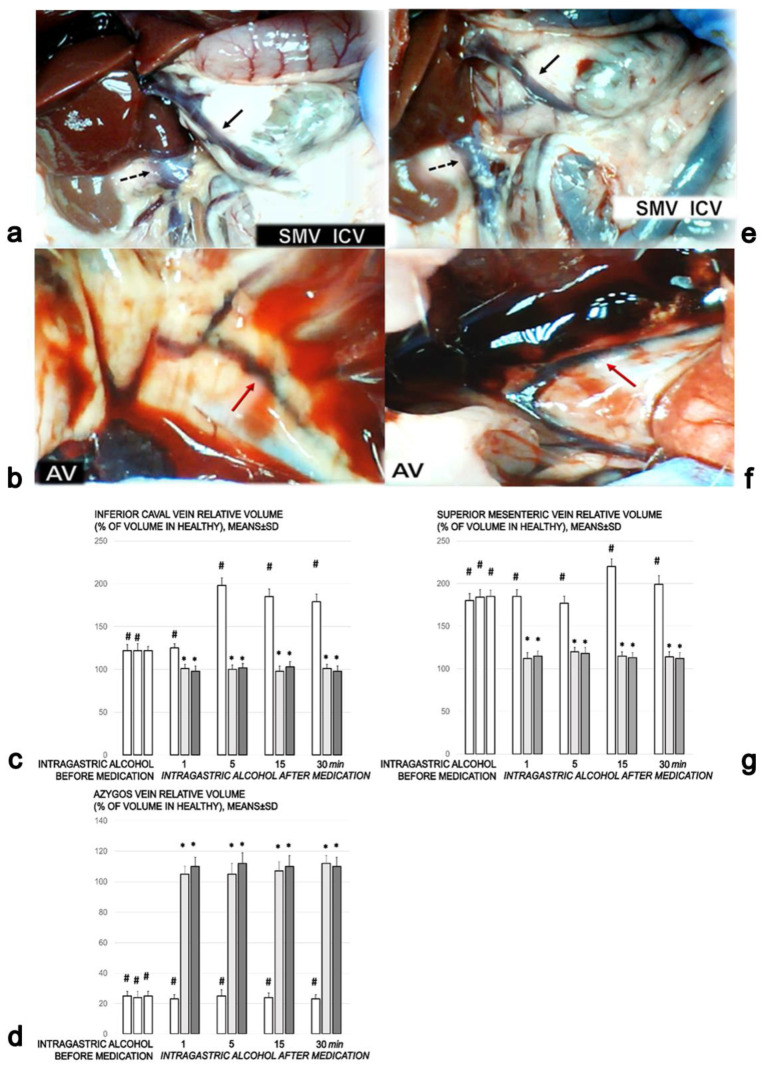
Vascular presentation. Gross presentation of the vessels (**a**,**b**) in saline-treated rats. *a.* Superior mesenteric vein (full black arrow) and inferior caval vein (dashed black arrows) (SMV, ICV), congested (white letters) ((**a**), controls). (**b**). Azygos vein (red arrow) (AV), failed (white letters) ((**b**), controls) in rats that received 1 mL of 96% alcohol introduced directly into the stomach, and saline 5 mL/kg IP (**b**) (left) at 1 min following alcohol. Presentation at 30 min thereafter, before euthanasia (camera attached to a VMS-004 Discovery Deluxe USB microscope (Veho, Dayton, OH, USA)). (**c**–**e**). Inferior caval vein (**c**), azygos vein (**d**) and superior mesenteric vein (**e**) relative volume (% of volume in healthy rats). Controls (white bars) who had 1 mL of 96% alcohol introduced in the stomach showed marked congestion of the inferior caval vein and superior mesenteric vein, and failed azygos vein presentation from the first minute until the end (1, 5, 15, and 30 min following therapy, which was given at 1 min following the introduction of 96% alcohol in the stomach (intragastric alcohol before medication)). The course was markedly counteracted with BPC 157 therapy (10 µg (light gray bars) or 10 ng/kg (dark gray bars)) IP). Six rats/group/interval. * *p* ˂ 0.05 at least vs. the corresponding control, # *p* ˂ 0.05 at least vs. normal healthy rats (100%). (**f**,**g**). Gross presentation of the vessels (**f**,**g**) in rats treated with BPC 157. (**f**). Azygos vein (red arrow) (AV), functioning (black letters). (**g**). Superior mesenteric vein (full black arrow) and inferior caval vein (dashed black arrows) (SMV, ICV), no congestion (black letters), in rats that received 1 mL of 96% alcohol introduced directly into the stomach, and BPC 157 10 ng/kg IP (**f**,**g**) (right) at 1 min following alcohol. Presentation at 30 min thereafter, before euthanasia (camera attached to a VMS-004 Discovery Deluxe USB microscope (Veho, Dayton, OH, USA)). The pictures obtained using BPC 157 10 ng/kg IP are representative of the observations for both doses.

A proportional change in the vessel was used to assess the development of peripheral vessel failure after intragastric alcohol administration (Figure 6a–g). There were rapidly induced peripheral vessel disturbances: the inferior vena cava and superior mesenteric vein volumes increased (Figure 6a,c,e) and the azygos vein completely failed, with a volume close to zero (Figure 6b,d). Therapy with BPC 157 rapidly attenuated these disturbances. The superior mesenteric vein and the inferior vena cava appeared normal (Figure 6c,e,g) and the complete failure of the azygos vein was reversed (Figure 6d,f). These benefits may have been due to the activated collateral pathways and the re-established blood flow.

### 3.7. Portal, Superior Mesenteric, Vena Caval, Abdominal Aortal and Superior Sagittal Sinus Pressure Recording

**Figure 7:**
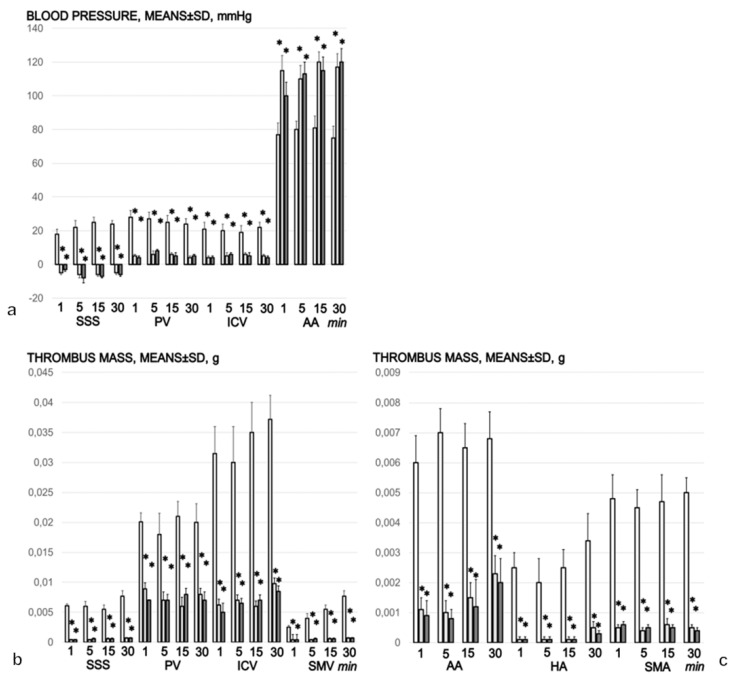
Blood pressure, mmHg (**a**), thrombus mass, g, assessed in veins (**b**) and arteries (**c**), the superior sagittal sinus (SSS), portal vein (PV), inferior caval vein (ICV), abdominal aorta (AA), superior mesenteric vein (SMV), superior mesenteric artery (SMA) and hepatic artery (HA) in the rats who had 1 mL of 96% alcohol introduced in the stomach, at 1, 5, 15, and 30 min following therapy. Therapy (BPC 157 10 µg (light gray bars) or 10 ng/kg (dark gray bars); saline (5 mL/kg (control, white bars)), IP) was administered at 1 min following the introduction of 96% alcohol in the stomach (upper). PQ-, QT-intervals and frequency (lower). The course was markedly counteracted with BPC 157 therapy. Six rats/group/interval. * *p* ˂ 0.05 at least vs. the corresponding control.

The intragastric administration of 96% alcohol led to severe portal and vena caval hypertension, with the former more pronounced than the latter, and aortal hypotension that persisted throughout the entire experimental period. In addition, the normal (negative) pressure in the superior sagittal sinus was increased (positive; Figure 7a). Therapy with BPC 157 rapidly resolved the severe portal and vena caval hypertension and aortal hypotension, and the increased (positive) pressure in the superior sagittal sinus was immediately restored to the negative pressure (Figure 7a).

### 3.8. Thrombosis

In the rats that received intragastric alcohol, thrombosis rapidly appeared, largely in the periphery, especially in the inferior vena cava, and then in the portal vein, the superior mesenteric vein (Figure 7b), the hepatic and superior mesenteric artery and the abdominal aorta (Figure 7c). It eventually progressed centrally and was visible in the superior sagittal sinus (Figure 7b). Treatment with BPC 157 markedly counteracted and reversed the thrombosis presentation (Figure 7b,c).

### 3.9. Brain Damage

**Figure 8:**
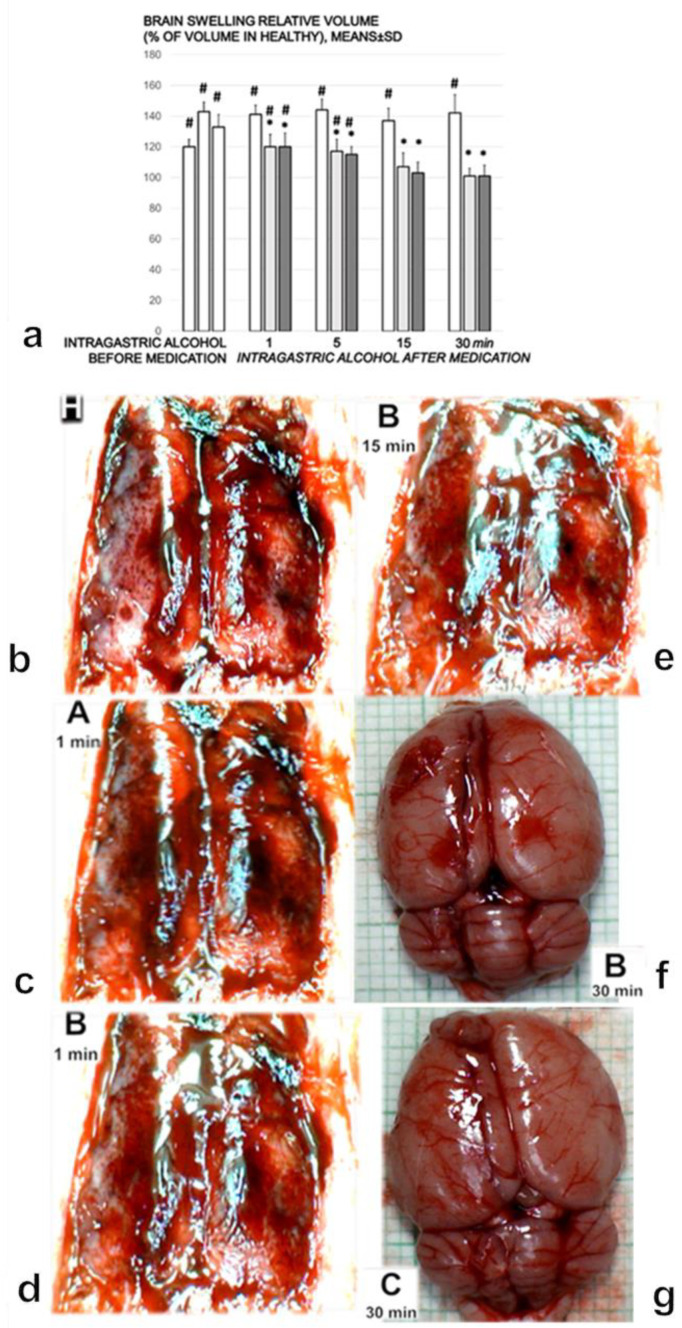
(**a**). Brain swelling relative volume (% of volume in healthy). Controls (white bars) with 1 mL of 96% alcohol introduced in the stomach showed increased swelling of the brain from the first minute until the end (1, 5, 15, and 30 min following therapy, which was given at 1 min following the introduction of 96% alcohol in the stomach (intragastric alcohol before medication)). The course was markedly counteracted with BPC 157 therapy (10 µg (light gray bars) or 10 ng/kg (dark gray bars)) IP). Six rats/group/interval. * *p* ˂ 0.05 at least vs. the corresponding control, # *p* ˂ 0.05 at least vs. normal healthy rats (100%). (**b**–**e**). When given to healthy rats (healthy brain presentation (H)), intragastric alcohol (A) induced brain swelling and BPC 157 (B) induced the reversal of the brain swelling, as per the timeline. One mL of 96% alcohol introduced directly into the rats’ stomachs, BPC 157 10 ng/kg IP at 1 min following alcohol. Brain presentation as follows: **b**. Healthy, (H), before the introduction of alcohol. (**c**). At 1 min following alcohol, but before therapy (A 1 min). (**d**). At 1 min following BPC 157 (B 1 min). (**e**). At 15 min following BPC 157 (B 15 min), (camera attached to a VMS-004 Discovery Deluxe USB microscope (Veho, Dayton, OH, USA)). The pictures obtained using BPC 157 10 ng/kg IP are representative of the observations for both doses. (**f**,**g**). Gross presentation of the brain in rats that received 1 mL of 96% alcohol introduced directly into the stomach, BPC 157 10 ng/kg IP (**f**) or saline 5 mL/kg IP (**g**) at 1 min following alcohol. Presentation at 30 min thereafter (BPC-157-treated rat, B 30 min (**f**), saline (control)-rat, C 30 min (**g**)), immediately after euthanasia (camera attached to a VMS-004 Discovery Deluxe USB microscope (Veho, Dayton, OH, USA)).

The rats without BPC 157 treatment exhibited brain swelling (Figure 8a,c,g). Without this counteraction (Figure 8a,c,g), there was a > 120% increase in the brain volume relative to the brain surface area compared with healthy rats (Figure 8a,g). In contrast to this intragastric alcohol-induced brain swelling (Figure 8c), the prominent effects of BPC 157 application appeared quite rapidly (Figure 8a,d–f).

**Figure 9:**
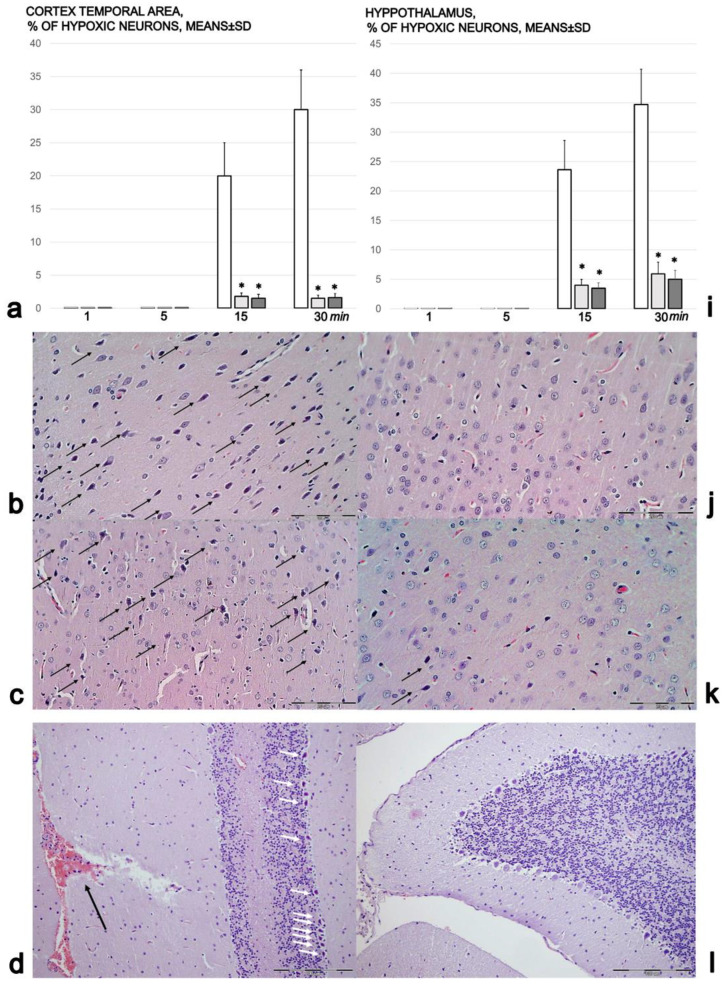

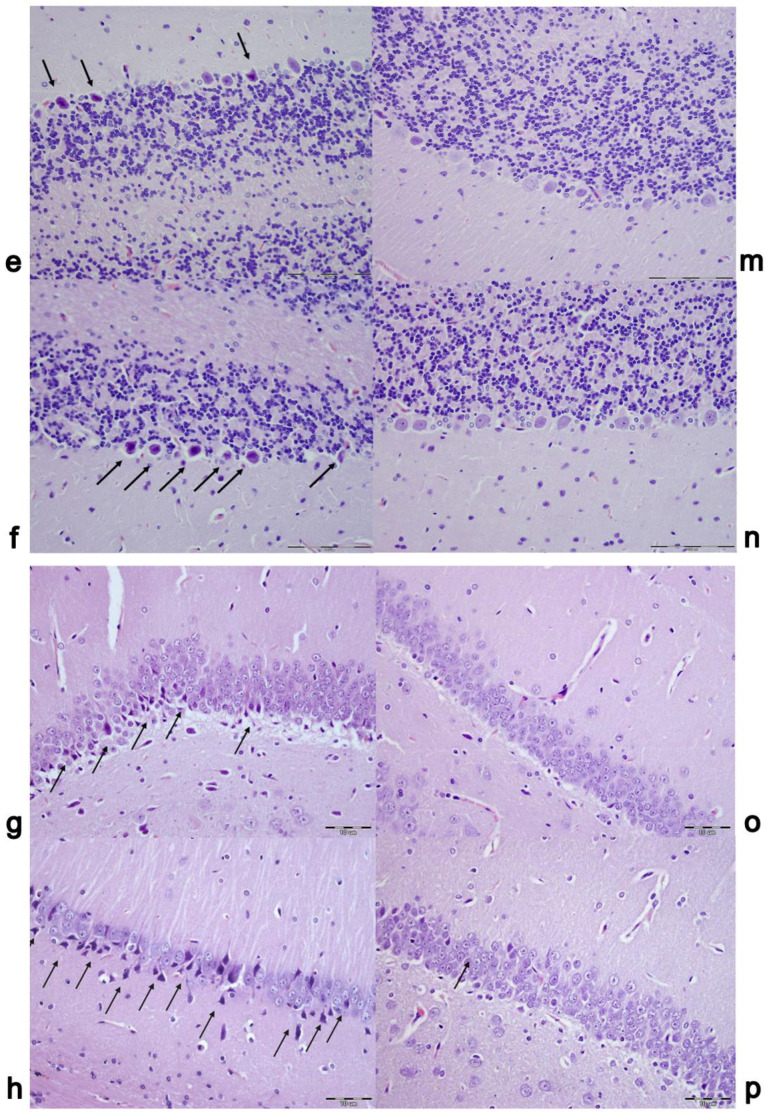
Brain lesions. Brain lesion (cortex temporal area, hypothalamus, (% of hypoxic neurons)) assessment and microscopic illustration of neuropathological changes in the cerebral cortex, cerebellar cortex, and hippocampus. (**a**). Brain lesion assessment (cortex temporal area (**a**), (% of hypoxic neurons)), in the rats that had 1 mL of 96% alcohol introduced in the stomach, at 1, 5, 15, and 30 min following therapy. Therapy (BPC 157 (10 µg (light gray bars) or 10 ng/kg (dark gray bars); saline (5 mL/kg (control, white bars)), IP) was administered at 1 min following the introduction of 96% alcohol in the stomach. The course was markedly counteracted with BPC 157 therapy. Six rats/group/interval. * *p* ˂ 0.05 at least vs. the corresponding control. (**b**–**h**). In controls (**b**–**h**), neuropathological changes in the cerebral cortex presented marked karyopyknosis of pyramidal cells in the cerebral cortex (arrows) (b (15 min), c (30 min)) and vascular congestion with edema (pericellular and perivascular empty zones) (HE, x400, scale bar 200 µm). Neuropathological changes in the cerebellar cortex in the controls presented with marked vascular congestion, edema and intracerebellar hemorrhage (black arrow) as well as degeneration of Purkinje cells in the cerebellar cortex (**d** (30 min), white arrows) (HE, ×200, scale bar 200 µm), marked karyopyknosis/degeneration of Purkinje cells in the cerebellar cortex (arrows) (**e** (15 min), **f** (30 min)) and vascular congestion with edema (HE, x400, scale bar 200 µm). The neuropathological changes in the hippocampus in the controls (**g**,**h**) were edema, congestion and karyopyknosis of neural cells in the hippocampus (arrows) (**g** (15 min), **h** (30 min)) (HE, x400, scale bar 200 µm). (**i**). Brain lesion assessment (hypothalamus (**i**), (% of hypoxic neurons)), in the rats that had 1 mL of 96% alcohol introduced in the stomach, at 1, 5, 15, and 30 min following therapy. Therapy (BPC 157 (10 µg (light gray bars) or 10 ng/kg (dark gray bars); saline (5 mL/kg (control, white bars)), IP) was administered at 1 min following the introduction of 96% alcohol in the stomach. The course was markedly counteracted with BPC 157 therapy. Six rats/group/interval. * *p* ˂ 0.05 at least vs. the corresponding control. (**j**–**p**) BPC 157 presentation. By comparison (**j**–**p**), rats treated with BPC 157 showed no changes in the cerebral cortex (**j** (15 min), and less than five karyopyknotic pyramidal cells/0.25 mm^2^ (**k** (30 min)) (HE, ×400, scale bar 200 µm). The rats treated with BPC 157 showed no described changes in the cerebellar cortex (**l** (30 min)) (HE, ×200, scale bar 200 µm), and a normal cerebellar cortex (**m** (15 min), **n** (30 min)); (HE, x400, scale bar 200 µm). They showed a normal hippocampus (**o** (15 min), **p** (30 min)) (HE, ×400, scale bar 200 µm). The pictures obtained using BPC 157 10 ng/kg IP are representative of the observations for both doses.

Furthermore, microscopically, the course was markedly counteracted with BPC 157 therapy (Figure 9a–p). The rats treated with saline after intragastric alcohol presented significant lesions in the cortex (Figure 9a–f) and hippocampus (Figure 9g–i). By contrast, the rats that received BPC 157 presented a structurally normal cortex (Figure 9a,j–n) and hippocampus (Figure 9i,o,p). The rats treated with saline after intragastric alcohol exhibited brain edema after 1 and 5 min, with vascular congestion. Furthermore, after 15 and 30 min, these rats showed generalized congestion, edema, and intracerebral hemorrhage, with degenerative changes in the cerebral and cerebellar neurons indicating toxic changes created by the ethanol. Regularly, the rats treated with BPC 157 after intragastric alcohol administration showed no cerebral or cerebellar tissue changes, with only mild congestion after 1 and 5 min. After 30 min, the rats showed minimal edema, with no degenerative changes to the cerebral, cerebellar and dark line hippocampus neurons. Within the 15–30 min period, the number of ‘dark’ neurons in the temporal cortex and in the hippocampus was markedly increased in the control group. Indeed, ‘dark’ neurons represented one fifth (temporal cortex) or of more (hippocampus) of the total neurons. By contrast, there was only a very small percentage of ‘dark’ neurons in the rats treated with BPC 157 (Figure 9a,i).

### 3.10. Gene Expression Analysis, eNOS, mTOR, and VGEFa

**Figure 10:**
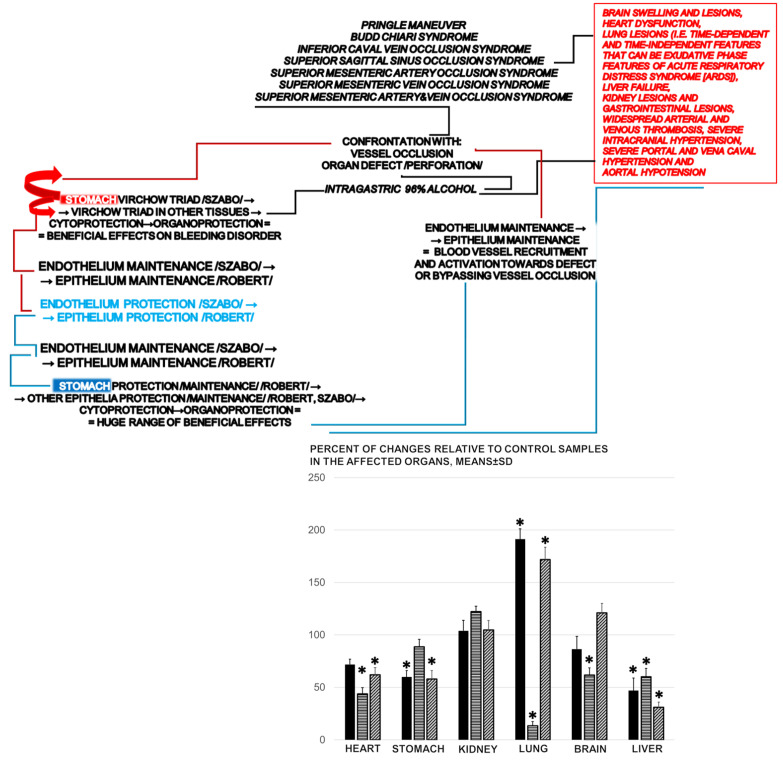
Summarizing the essential epithelium and endothelium protection interplay known in Robert and Szabo’s cytoprotection, and the role of the stable pentadecapeptide BPC 157 as a likely mediator, we suggest that BPC 157 may be useful as cytoprotective therapy. Hopefully, the huge theoretical importance of the all aspects of the cytoprotection concept may finally be realized in practice. Conceptually, taking intragastric alcohol as Virchow triad occlusive syndrome, there is a new point (bypassed occluded or ruptured vessel, equation endothelium maintenance → epithelium maintenance = blood vessel recruitment and activation towards defect or bypassing vessel occlusion), the recruitment of collateral blood vessels to compensate for vessel occlusion and reestablish blood flow. The BPC 157 counteracted various venous occlusion-induced syndromes, inferior caval vein syndrome, superior sagittal sinus occlusion syndrome, superior mesenteric artery occlusion syndrome, superior mesenteric vein occlusion syndrome, superior mesenteric artery and vein occlusion syndrome, Pringle maneuver ischemia, reperfusion, and Budd–Chiari syndrome in rats. The activation of the alternative collateral pathways to bypass occlusion, and the reestablishment of alternative blood flow, resulted in the counteraction of the full range of consequent perilous syndromes. Thus, intragastric alcohol application-induced syndrome comparable to the effects of major vessel occlusion was also counteracted with the application of BPC 157. As an illustration of the complexity of these beneficial effects, when assessed at 5 min following intragastric alcohol challenge, *eNOS*, *mTOR*, and *VGEFa* expression showed a particular presentation. Gene expression analysis: *eNOS* (black bars), *mTOR* (horizontally dashed white bars), and *VGEFa* (vertically dashed gray bars) at 5 min following intragastric alcohol challenge. Percentage of changes relative to control samples in the affected organs (heart, stomach, kidney, lung, brain and liver). Therapy (BPC 157 (10 ng/kg); saline (5 mL/kg (control)), IP) was administered at 1 min following the introduction of 96% alcohol in the stomach. Six rats/group/interval. * *p* ˂ 0.05, at least vs. the corresponding control.

As an illustration the complexity of the beneficial effect, when assessed at 5 min following the intragastric introduction of alcohol, *eNOS*, *mTOR*, and *VGEFa* expression showed a particular presentation (Figure 10). It is likely that this particular presentation depended on the affected organ, and the particular processes initiated while all of these organs (the stomach, and then the brain, heart, lung, liver and kidneys) were fairly protected, grossly and microscopically, by the administration of BPC 157. The *eNOS* specificity demonstrated a decreased expression in the stomach and liver, an increased expression in the lung, while the brain, heart, and kidneys not affected. The *mTOR* specificity demonstrated a decreased expression in the heart, lung, brain and liver, and the stomach and kidneys were not affected. The *VGEFa* specificity demonstrated a decreased expression in the heart, stomach and liver, an increased expression in the lung, and the brain and kidneys were not affected.

### 3.11. Summary

In summary, we found that in rats subjected to intragastric alcohol administration, BPC 157 therapy counteracted the gastric lesions as well as the peripheral and central deficits. There was rapid resolution that adequately reversed the anatomical imbalance of venous drainage and improved the peripheral and central deficits. The outcomes were the attenuation of thrombosis and the counteraction of brain, heart, lung, liver, kidneys, gastrointestinal lesions. In addition, gene expression analysis showed a particular presentation of the *eNOS*, *mTOR*, and *VGEFa* in the heart, stomach, kidneys, lung, brain and liver (see Figure 10 as summarized background).

## 4. Discussion

Of note, the prototypical model of cytoprotection, namely Robert’s absolute alcohol intragastric application, is usually described in terms of epithelial and endothelial injury and thrombosis [1,2,3]. However, our study was the first essential extension to describe the full intragastric alcohol application-induced occlusive syndrome, which includes peripheral and central dysfunction resembling the deficits that have been described in rats with major vessel occlusion [11,12,13,14,15,16,17]. We claimed that in both noxious events, the intragastric introduction of alcohol and major vessel occlusion [11,12,13,14,15,16,17], an effective therapy is the activation of bypass collateral pathways, which is key to cytoprotection studies and the screening the cytoprotective activity of agents. The administration of BPC 157 has been shown to counteract the deficits induced by the occlusion of major vessels [11,12,13,14,15,16,17]. In this study, likely in the same way, BPC 157 counteracted the gastric lesions, peripheral (portal and vena caval hypertension and aortal hypotension) and central (brain swelling and intracranial hypertension) disturbances and organ lesions induced by intragastric alcohol administration. In addition, this may have taken place along with the activation of the specific molecular pathways, i.e., eNOS, mTOR and VGEFa, known to interact with alcohol and the application of BPC 157 [7,10,58,59] (Figure 10).

Intragastric alcohol application-induced syndrome comparable to the effects of major vessel occlusion follows the initial insult to the stomach. Marked gastric lesions alongside Virchow’s triad, inside and outside the stomach, appear with the same characteristics as the previously mentioned occlusive syndromes produced by the particular occlusion of one or two major vessels [11,12,13,14,15,16,17]. Since BPC 157 exerts beneficial effects against permanent vessel occlusion syndromes by activating collateral bypassing loops [11,12,13,14,15,16,17], the benefits of BPC 157 against intragastric alcohol application likely involve a similar activation of the relevant collateral pathway(s). As a likely rescue pathway, we identified the activated azygos vein pathway and the inferior vena cava–azygos vein–left superior vena cava pathway. In the rats treated with saline after intragastric alcohol, the azygos vein had completely failed, a phenomenon that also occurs in the rat model of Budd–Chiari syndrome [14] and central venous occlusion [17]. In the intragastric alcohol-treated rats administered BPC 157, the inferior vena cava and superior mesenteric vein congestion was reversed, reflecting the elimination of the otherwise severe vena caval and portal hypertension. Thus, as has been noted in vessel occlusion syndromes [11,12,13,14,15,16,17], the principle of endothelium maintenance → epithelium maintenance [1,8] was upgraded to endothelium maintenance → epithelium maintenance = blood vessel recruitment and activation (‘running’) towards the site of injury, or bypassing occlusion via alternative pathways [5,6] in the intragastric alcohol-treated rats.

There may be, however, other activated bypassing loops. For example, in the rats with an occluded superior sagittal sinus, we identified central shunts through the ophthalmic vein, the angularis vein, the facial anterior and posterior veins, and the facial vein, as well as the superior cerebral veins, the superior and inferior sinus cavernosus and sinus petrosus, the sinus transversus, the external jugular vein, the subclavian vein and the superior vena cava [17]. Thereby, with BPC 157 therapy delivered topically on the swollen brain, intraperitoneally or intragastrically, there was rapid attenuation of the brain swelling [17]. Therapy with BPC 157 rapidly eliminated the increased pressure in the superior sagittal sinus, the severe portal and vena caval hypertension and aortal hypotension, it quickly recruited collateral vessels, abrogated venous and arterial thrombosis, and it helped in the recovery of the organ lesions [11,12,13,14,15,16,17]. Evidently, BPC 157 offers therapeutic benefits in the resolution of damage due to intragastric alcohol or peripheral [11,12,13,14,15,16] or central [17] vessel occlusion.

It appears that after the intragastric introduction of alcohol, BPC 157 therapy offers strong beneficial effects to the stomach and brain regions, including the cerebral and cerebellar cortices, the hypothalamus, the thalamus and the hippocampus. For example, BPC 157 attenuates intracranial hypertension and thereby counteracts edema and intracerebral hemorrhage due to intragastric alcohol, which lead to hypoxic/dark neurons and degenerative changes in the cerebral and cerebellar neurons. It also helps to maintain (negative) blood pressure in the superior sagittal sinus [17]. The shunts activated by BPC 157 provide for a well-functioning and rapidly established alternative equilibrium to compensate for the injurious course of intragastric alcohol administration, similarly to central venous occlusion [17], as well as peripheral superior mesenteric artery or superior mesenteric artery and vein occlusion [13,15,16]. If this resolution of impaired venous drainage does not occur centrally, the harmful build-up of venous blood leads to venous and intracranial hypertension [65,66,67,68,69,70]. Indeed, this rise occurred in essentially the same high range in the controls with the intragastric alcohol and a permanently occluded superior sagittal sinus [17], and to a slightly lesser extent in the controls with an occluded superior mesenteric artery or an occluded superior mesenteric artery and vein [13,15,16]. Of note, this particular vascular network organization, which BPC 157 induces to maintain undisturbed intracranial pressure (superior sagittal sinus), may work against further worsening that could appear with either peripheral or central challenges.

As has been described in previous studies [11,12,13,14,15,16,17], the lack of thrombosis, or at least the marked attenuation of thrombosis, is key to the elimination/attenuation of general stasis, as well as the resolution of Virchow’s triad [11,12,13,14,15,16,17,36,37,38,39] and the re-establishment of blood flow. Without therapy, general stasis—a large volume of blood trapped in the damaged stomach, the CNS, and the portal and vena caval tributaries—may perpetuate brain and heart ischemia and lead to the rapid appearance of thrombosis. The intragastric alcohol-treated rats showed disturbances in all examined vessels, similarly to the results of previous studies examining major vessel occlusion [11,12,13,14,15,16,17]. These deficits likely reflect a low-flow state caused by cardiac dysfunction or severe volume depletion [68]. Without therapy, the intragastric alcohol-treated rats exhibited considerable heart and lung disturbances: a prolonged QT interval, subendocardial infarct, heart dilatation, congestion and hemorrhage in the lung parenchyma, resembling the exudative features of ARDS. These have all been observed in rats subjected to vessel occlusion [11,12,13,14,15,16,17], and collectively these findings implicate the heart and lungs as additional prime targets. Subsequently, there was liver and kidney failure, progressive congestion and extensive gastric hemorrhagic lesion, along with prominent portal and vena caval hypertension. Thus, the intragastric alcohol course mimics the supporting associations also noted in patients [21,22,23,24] and in previous BPC 157 studies, particularly studies involving alcohol, in which BPC 157 consistently counteracted gastric lesions induced by 96% alcohol [31,71,72,73,74].

Alcohol affects thrombocyte function [21]: the acute ingestion of a relatively large but tolerable dose of alcohol transiently enhances thromboxane-mediated platelet activation [22]. The same goes for atrial fibrillation, supraventricular tachycardia, and ventricular tachycardia, and even lethal arrhythmias in patients with myocardial infarction, contractile dysfunction leading to heart failure, stroke, and increased risk of cardiac death [23]. Alcoholic hepatitis and acute or chronic liver failure follow acute alcohol intoxication [23]. There is also a strong, dose-dependent association between the number of alcoholic drinks consumed per day and acute lung injury and ARDS [23]. In addition to its previously described effects on vessel recruitment [11,12,13,14,15,16,17,36,37,38,39] BPC 157 counteracts chronic alcohol consumption-induced stomach lesions, liver failure and portal hypertension [75,76], acute alcohol (4 g/kg intraperitoneally) intoxication, and chronic (withdrawal) alcohol intoxication [74,77]. When administered before or after ethanol, BPC 157 acts as an alcohol antagonist and provides extended cytoprotective effects against the anesthesia, hypothermia, increased ethanol blood values and fatality [77,78], opposes thiopental anesthesia [79], and counteracts various encephalopathies [80,81,82,83,84,85] in rats. Similarly to its counteraction of encephalopathies [80,81,82,83,84,85], BPC 157 likely counteracts multiple pathologies in the gastrointestinal tract and liver [80,81,82,83,84,85]. Moreover, BPC 157 counteracts various arrhythmias [86,87,88,89,90]. In particular, BPC 157 therapy normalizes the QTc duration in rats treated with neuroleptics, and it prevents and improves chronic heart failure [88,89]. It also improves lung pathology (i.e., pulmonary hypertension syndrome in chickens [91], monocrotaline pulmonary hypertension in rats [92]) and intratracheal HCl instillation-induced lung lesions in rats [93].

In conclusion, in the intragastric alcohol-treated rats, alcohol caused an initial endothelial injury that quickly spread and mimicked the symptoms observed when major vessels are permanently occluded in animal models [11,12,13,14,15,16,17]. For therapy, the cytoprotective equation, endothelium maintenance → epithelium maintenance = blood vessel recruitment and activation towards defect or bypassing vessel occlusion [11,12,13,14,15,16,17,36,37,38,39], is crucial. At least at the general level, Robert’s prototypical intragastric alcohol-induced gastric lesion presents as an occlusive syndrome with peripheral and central defects. Therapy with the stable pentadecapeptide BPC 157 promoted recovery that was similar to that which has been observed in rat models subjected to major vessel occlusion and then administered BPC 157 [11,12,13,14,15,16,17,36,37,38,39]. Of note, the doses of BPC 157 used in this study were similar to those used in the previous studies that suggested that BPC 157 may exert its effects through the NO system [5,6,10], perhaps by inducing NO release [72,94]. BPC 157 may also counteract hypertension and pro-thrombotic effects by modulating the action of L-NAME [33,34,35,72], as well as hypotension and anti-thrombotic effects by modulating the action of L-arginine [33,34,35,72]. To improve vasomotor tone, BPC 157 could activate the Src–Caveolin-1–eNOS pathway [48]. There is evidence that BPC 157 modulates the prostaglandin system [5,6,9] to counteract the adverse effects of NSAIDs, cyclooxygenase (COX)-1, and COX-2 blockers [80,81,82,83,84,85], due to its particular effect as a membrane stabilizer that counteracts leaky gut syndrome [46]. Moreover, it prevents the development of arthritis and cures already established lesions in rat models [95]. These effects are likely due to the role of BPC 157 as a bypassing key [5,6].

In addition, the study of such beneficial effects as the particular activation of the collateral pathways reliant on the injurious occlusion [11,12,13,14,15,16,17], along with the activated molecular pathways (at least, *eNOS*, *mTOR* and *VGFRa* in the stomach, brain, heart, lung, liver and kidneys) [40,46,47,48,49,50,51,52,53,54], should be combined with the extensive studies that have been performed on how BPC 157 exerts these specific effects. For instance, a study of mitigated leaky gut syndrome revealed that BPC 157 acts as a stabilizer of the cellular junction, via increasing tight junction protein ZO-1 expression and transepithelial resistance [46]. There were inhibited mRNA of inflammatory mediators (iNOS, IL-6, IFNγ and TNF-α), an increased expression of HSP 70 and 90, and antioxidant proteins, such as HO-1, NQO-1, glutathione reductase, glutathione peroxidase 2 and GST-pi [46]. Of note, the antioxidant effect of BPC 157 [55,56,57] occurs in both ischemic and reperfusion conditions in the various tissues (i.e., colon, duodenum, cecum, liver and veins) and plasma [11,12,13,15,16,36,37,38,39].

In one study, BPC 157 affected *Egr*, *Nos*, *Srf*, *Vegfr*, *Akt1*, *Plcɣ* and *Kras* gene expression in the vessel that provided an alternative operating pathway (i.e., the left ovarian vein as the key to the infrarenal occlusion-induced inferior vena cava syndrome in rats) [11]. In the hippocampus, BPC 157 strongly elevated *Egr1*, *Akt1*, *Kras*, *Src*, *Foxo*, *Srf*, *Vegfr2*, *Nos3* and *Nos1* expression and decreased *Nos2* and *Nfkb* expression; these changes may indicate how BPC 157 exerts its effects [40]. Therefore, in the rats who were received intragastric alcohol, BPC 157 maintained the integrity of the affected organs; the distinctive, evidenced changes to *eNOS*, *mTOR* and *VGFRa* expression may illustrate particular points at which BPC 157 therapy worked simultaneously in each of the organs. An illustrative example may be the simultaneous increase (*eNOS*, *VGFRa* expression) and decrease (*mTOR* expression) in the lung. Interestingly, we noted that *eNOS*, *mTOR* and *VGFRa* expression occurred together in the liver (decrease), and in the kidneys (no change). Overall, BPC 157 can induce and interact with a host of molecules to exert beneficial effects. The extent of the benefits of BPC 157, and the mechanisms by which it exerts these benefits, remain to be fully explored.

As a final note, it is true that animal studies *per se*, especially cytoprotection studies, must be interpreted with caution [96]. In addition, there is a relative paucity of BPC 157 clinical data [5,6]. However, BPC 157 has proved to be efficacious in the treatment of ulcerative colitis [5,6], in both clinical settings [97,98] and in experimental animal models [36,80,81,82,83,84,85,99,100], as well as in complications (for review, see [101]). An important point regarding the application of cytoprotection in practice in various species [100] is the very safe profile of BPC (the lethal dose (LD1) could be not achieved) [7,45]), a point recently confirmed in a large study by Xu et al. [102]. In this context, and also for practical purposes, given that the therapeutic effects speak for themselves, especially in cytoprotection studies [1,2,3,8], animal models offer indispensable substantiation of the actions and principles by which BPC 157 exerts its benefits in multiple settings, including alcohol intoxication.

## Figures and Tables

**Table 1:** Selected genes and TaqMan Assays specifications.

Gene Symbol	Synonyms	Gene Name	TaqMan Assay ID	NCBI Reference Sequence	Amplicon Length (bp)
*Actb*		Actin, beta	Rn00667869_m1	NM_031144.3	91
*Nos3*	cNOS, eNos	Nitric oxide synthase 3	Rn02132634_s1	NM_021838.2	117
*Mtor*	Frap1, RAFT1, RAPT1	Mechanistic target of rapamycin kinase	Rn00693900_m1	NM_019906.1	70
*Vegfa*	VEGF-A, VPF	Vascular endothelial growth factor A	Rn01511601_m1	NM_001110333.2	69
